# Erratum: Closed-form solutions and scaling laws for Kerr frequency combs

**DOI:** 10.1038/srep26920

**Published:** 2016-06-10

**Authors:** William H. Renninger, Peter T. Rakich

Scientific Reports 6: Article number: 2474210.1038/srep24742; published online: 04252016; updated: 06102016.

This Article contains errors.

The title of Figure 3,

“Solution dependence the normalized drive strength.”

should read:

“Solution dependence on the normalized drive strength.”

In the legend of Figure 4,

“*γ* = 1(*Wm*)”

should read:

“*γ* = 1/(*Wm*)”

